# Tool for evaluating research implementation challenges: A sense-making protocol for addressing implementation challenges in complex research settings

**DOI:** 10.1186/1748-5908-8-2

**Published:** 2013-01-02

**Authors:** Kelly M Simpson, Kristie Porter, Eleanor S McConnell, Cathleen Colón-Emeric, Kathryn A Daily, Alyson Stalzer, Ruth A Anderson

**Affiliations:** 1 Durham Veteran Affairs Medical Center, GRECC, 508 Fulton St., Durham, NC, 27705, USA; 2 Duke University, School of Nursing, 307 Trent Dr., Durham, NC, 27710, USA; 3 KayeM, Inc, 24 Tinsbury Place, Durham, NC, 27713, USA

**Keywords:** Long-term care, Complexity science, Nursing homes, Implementation research, Intervention research, Research design, Staff intervention, Sense-making, Research fidelity

## Abstract

**Background:**

Many challenges arise in complex organizational interventions that threaten research integrity. This article describes a Tool for Evaluating Research Implementation Challenges (TECH), developed using a complexity science framework to assist research teams in assessing and managing these challenges.

**Methods:**

During the implementation of a multi-site, randomized controlled trial (RCT) of organizational interventions to reduce resident falls in eight nursing homes, we inductively developed, and later codified the TECH. The TECH was developed through processes that emerged from interactions among research team members and nursing home staff participants, including a purposive use of complexity science principles.

**Results:**

The TECH provided a structure to assess challenges systematically, consider their potential impact on intervention feasibility and fidelity, and determine actions to take. We codified the process into an algorithm that can be adopted or adapted for other research projects. We present selected examples of the use of the TECH that are relevant to many complex interventions.

**Conclusions:**

Complexity theory provides a useful lens through which research procedures can be developed to address implementation challenges that emerge from complex organizations and research designs. Sense-making is a group process in which diverse members interpret challenges when available information is ambiguous; the groups’ interpretations provide cues for taking action. Sense-making facilitates the creation of safe environments for generating innovative solutions that balance research integrity and practical issues. The challenges encountered during implementation of complex interventions are often unpredictable; however, adoption of a systematic process will allow investigators to address them in a consistent yet flexible manner, protecting fidelity. Research integrity is also protected by allowing for appropriate adaptations to intervention protocols that preserve the feasibility of ‘real world’ interventions.

## Background

Increasingly, scholars recognize that complex research interventions may be necessary for multifactorial problems within complex healthcare environments [1]. While easier to design and implement, health services interventions that narrowly focus on individuals or single work groups may not acknowledge the complex interdependencies between patients and healthcare staff at various levels of the organization and may therefore fail in real-world applications. Alternately, we propose that complex interventions will be more likely to result in significant and sustained changes. Thus, it is necessary to explore ways to overcome challenges to implementing research designs that test complex interventions.

In this article, we describe a protocol developed to address the research challenges arising during implementation of two complex interventions in healthcare organizations. We draw on a complexity science perspective [2,3] to interpret these experiences and codify a set of strategies that can be used in other complex interventions and research settings. Specifically, we propose a sense-making approach to managing implementation challenges for intervention research in complex settings. Sense-making is a group process in which diverse members interpret challenges when available information is ambiguous; the groups’ interpretations provide cues for taking action [4].

### The study example: CONNECT for quality

The CONNECT for Quality study tests two multi-component organizational interventions aimed at reducing falls in nursing homes. This study used a multi-site, randomized controlled design and was conducted in two separate pilot studies; one study in four community nursing homes one study in four Veterans Affairs (VA) Community Living Centers, referred to hereafter as nursing homes. One intervention, CONNECT, was an intensive educational intervention focused on increasing the number and quality of connections between nursing home staff at all levels to promote the staff’s capacity to implement evidence-based falls prevention practices. The second intervention was the Agency for Healthcare Research Quality toolkit for falls prevention, Falls Management Program [5], currently a gold standard for training nursing home staff about how to prevent resident falls. Detailed descriptions of these intervention protocols have been previously published [6].

The research designs for these studies were not developed in isolation from the participant population. The content for the CONNECT intervention was developed during a five-year case study in which we (the PIs RAA and CCE) led research using in-depth and extensive engagement in the field, recording observations and interviews. This study was followed by a year-long period in which we returned to participants in the population (locally and nationally) to discuss intervention content and implementation strategies, and we also conducted small pilot studies testing individual components of the intervention. Although we used a participatory approach to design the full intervention study, we still encountered challenges during study implementation. We believe challenges will arise no matter how the study is planned because we are working in complex adaptive systems (described next). Studies that plan in isolation from the participant population will likely experience more unexpected challenges than studies that work with participant populations in the study design, but all studies will have implementation challenges because of the nature of complex adaptive systems.

### Complex adaptive systems, complexity science, and implementation

The two complex interventions were delivered in nursing homes, a healthcare environment now considered by many to be a complex adaptive system [7]. Nursing homes are complex adaptive systems because they are comprised of a collection of individual agents (staff, residents, and families) with the freedom to act in ways that are not always totally predictable, and whose actions are interconnected; for example, one agent's actions change the context for other agents in the system [8]. Complexity science, the study of relationships between and among system agents and how they give rise to collective behaviors and outcomes [9], provides insight as to how these systems work. Key characteristics of complex adaptive systems are more fully defined in Table 1.

**Table 1:** Hallmarks of complex adaptive systems

**Hallmarks of complex adaptive systems**	**Definitions**	**Example from research setting**
Large Number of agents	Agents are system components. In healthcare settings, agents may be people (*e.g.*, physicians, patients, administrators), processes (*e.g.*, nursing processes), or functional units (*e.g.*, accounting), and organizations (*e.g.*, regulatory agency) [10].	Subjects, healthcare providers, research team members, administrators, regulators, funders
The agents are diverse	The more diverse the agents, the greater the likelihood of novel behavior [10].	Diverse training, background, expertise, experience
The agents are connected and interdependent	The number and quality of connections among agents. Interdependence ensures that the performance of any one individual is not the additive function of the actions of that agent. Agents interact and use each other’s knowledge and skills, and build on each other’s work products [11].	Frequent interactions for coordinating and implementing the protocol, regular study team meetings
Relationships among agents are non-linear and unpredictable	Patterns of the relationships between the agents do not directly reflect the inputs and outputs from the relationships [10].	Relationship between study team and setting administrator may facilitate or hinder protocol implementation
Agents interact with the environment and both co-evolve	Agents respond to the environment and/or other agents but the reciprocal environment and/or agent also change from the interaction, influencing how both develop [12].	Study team adapts intervention schedule based on clinical routine at the site. Site implements new routine to facilitate recruitment (*e.g.*, pizza at meeting)
The system’s future is linked to its past because of its history of co-evolution.	The history of an agent and its interactions shape its current and future state but does not preclude unpredictable transformation of a complex adaptive system at any given time [13].	Prior experience with research by the site may influence its implementation of current project
Agents self-organize	Agents interact and mutually adjust behaviors to meet demands of the environment. Through self-organization, new patterns of behavior emerge [14].	Researchers learn ways to be mobile—moving to locations to do the intervention rather than having staff come to the researchers
System dynamics lead to emergence of new forms or order which are not under centralized control	‘Patterns and processes that occur within the underlying networks play a major role in the emergence of system-wide features;’ (page, 623). These are discernible global patterns over which there is no centralized control [9].	Study subjects may be more or less likely to participate based on what other agents in the system are saying about the study; higher or lower site participation rates result

Specifically, complexity theory proposes that outcomes that emerge in complex adaptive systems are often non-linear and unpredictable. Interactions at the local level give rise to global system patterns that cannot be explained by individual actions of agents. The theory suggests therefore that one must consider the interplay between system agents when managing unpredictable events. We propose that multiple agents involved in implementing complex organizational interventions—such as the research team, protocol, and setting—also constitute a complex adaptive system. Complexity theory offers a way to understand complex adaptive systems and provides insight into research implementation in these settings [2]. Strategies for addressing the challenges of research implementation develop, or emerge, through interactions between the research protocol, research team, and site.

In the CONNECT study, multiple layers of complexity were present. First, the full research team, which bridged two pilot studies and two organizations, was diverse, comprising 27 individuals with different professional backgrounds (*e.g.*, nursing, medicine, public health, social sciences, business, statistics), educational levels (*e.g.*, baccalaureate, masters, PhD/MD), and various levels of experience working in nursing homes (*e.g.*, from none to over 25 years). We had a smaller core implementation team of about 10 people, however, including the principal investigators, co-investigators, project directors, and research interventionists, who met in team meetings weekly and implemented the research protocol. Second, the research protocol included two complex multi-faceted interventions with a combined total of 13 components. Third, complexity related to protocol implementation was increased because of the need to accommodate the unique features of eight sites and the requirements of five institutional review boards. The nursing home study sites were themselves complex adaptive systems [2]. Heterogeneity was the norm both within and between individual nursing homes, including in the number of staff (range 109–229), professional training and experience (*e.g.*, occupational and physical therapy, social work, dietary, medicine, nursing, housekeeping, and support staff), and work cultures (*e.g.*, strong chain of command, punitive management practices). We interacted with people in all disciplines and all levels of the organization during the intervention adding to the potential for non-linear, unpredictable, and emergent outcomes.

The interactions between these diverse agents in the CONNECT study resulted in a number of research implementation challenges that required modification of study procedures, strategy, and/or protocols. These adjustments were needed to support successful implementation while maintaining the integrity of the research design.

### Implementation challenges in complex research settings

Prior literature has described a number of challenges that are common when conducting research in complex settings such as nursing homes. Specific to nursing homes, these challenges include staffing issues (turnover, absenteeism, unstable schedules, nursing staff ratios) [15,16], staff mistrust of the research process [17], and organizational climate and culture [17]. Other external variables may include profit status, chain affiliation, and regulatory oversight [15]. During the implementation process, our research team experienced a number of challenges related to these issues, including nursing home staff who were unfamiliar with the research process, frequent scheduling changes, respondent burden related to the intensity of the protocol (multiple measures, multiple waves of data collection), and research staff who were new to the nursing home environment.

In addition to these oft-noted implementation issues, our research team encountered several challenges that might also be applicable to intervention research in any complex healthcare setting. In Table 2, we grouped examples of the challenges into three types: those arising from the research site; those arising to the protocol itself; and those arising from the research team. We outline broad categories of challenges that arose in our research, recognizing that most will be common across research settings, and indicate the degree to which each challenge was deemed a threat to research integrity and then identify the type of fidelity that might be impacted. In the last column, we provide examples of solution strategies that we developed in three areas, including research design, research staffing, and research implementation. This is not an exhaustive list of challenges assessed and resolved using our process, but it exemplifies the types of challenges that might arise from different aspects of the research (site, protocol, or team). In the next section we discuss how we arrived at the solution strategies noted in Table 2, and why we believe the Tool for Evaluating Research Implementation Challenges (TECH), which we introduce below, is a sound approach for addressing challenges in complex organizational research settings.

**Table 2:** Implementation challenges, examples, threats to research integrity, and strategies for overcoming

**Challenge**	**Example**	**Threat to research integrity**	**Strategies (RD = research design, RS = research staffing, RI = research implementation)**
**Research Setting**			
Competing clinical demands on staff	Staff unable to leave floor to attend study activities	Intervention dose, effectiveness	RD: Design intervention with flexibility (group or individual, classroom or on nursing station, online or paper materials), combine activities when possible.
			RS: Pairs of research staff may be able to complete intervention and data collection more efficiently.
			RI: Identify and use times of day and venues most convenient for staff. Clearly explain study time requirements and obtain commitment from administration during nursing home recruitment. Use advisory board of site employees to inform implementation.
	Unable to identify staff for additional training for sustaining intervention after study ends	Sustainability	RI: Include nursing home management in study early to garner enthusiasm and support for release time. Identify and use meaningful incentives. Do not select staff a priori based on their role, but await understanding of particular nursing homes setting and the emergence of a champion with appropriate skills.
Nursing home staff turnover, schedule changes, and absenteeism	Frequent staff list changes, changes in shift	Drop-out rates	RD: Include plan for adding new staff participants to study prospectively, when possible. Power study appropriate for dropout rates, measure turnover and include in analysis plan.
		Intervention dose, effectiveness	
			RI: Include plan for study procedures to occur during new staff orientation.
			RS: Schedule research staff for all shifts during data collection.
Nursing home staff diversity	Variety of literacy levels, educational backgrounds, and primary language makes study intervention and data collection challenging	Data validity	RD: Include a variety of materials targeted for different staffing types at appropriate educational levels. Use multiple delivery methods such as written, oral, storytelling. Develop formal methods of assessing intervention receipt such as skill enactment.
		Recruitment and Retention	
Nursing home work culture	Staff in facilities with hierarchical, punitive work cultures less willing to participate	Recruitment and Retention	Provide locked drop boxes for surveys and consents. Provide tear-off cover pages on surveys so that staff can remove identifying information other than study number prior to return. Identify private areas for staff-researcher interactions.
**Research Protocol**			
Intensity of research work in nursing home	Researcher fatigue	Protocol fidelity	RI: Schedule one to two days a week for which the interventionist does not travel to the nursing home.
		Data collection errors	
			RS: Pair research staff in each home to provide breaks, flexibility.
Complex interventions with multiple components	Difficult to ensure that the interventions are delivered to all staff across multiple sites in similar dose and quality	Fidelity	RD: Include fidelity measurements and assessment in design.
			RI: Use detailed intervention manuals with interventionist training, monitoring and feedback protocols.
**Research Team**			
Lack of familiarity with the NH setting	Research staff alienate nursing home staff when they unknowingly interrupt key activities or exhibit ‘ignorance’ of setting	Recruitment	RS: Plan extensive training period with time spent in nursing home and with nursing home staff. Include readings and key points about setting in training materials. Pair more and less experienced staff together.
		Retention	
		Inefficiency	

## Methods

### Tool for evaluating research implementation challenges

Early in the implementation of our study we began using a process for sense-making that we have now codified as the TECH*.* Sense-making, which occurs naturally in well-functioning complex adaptive systems, is a group process in which diverse members interpret challenges for which available information is ambiguous; the interpretations provide cues for taking action [4]. The sense-making protocol outlines a process for the research team to systematically define challenges, assess their potential impact on intervention fidelity, and determine next actions. The TECH is a distinctive approach in that it is shaped by complexity theory, and it provides a general process to assess and interpret implementation issues and identify and evaluate solutions. The process alerts researchers to unexpected issues that might arise and provides a way to approach them while also considering fidelity of the research. It contributes to the implementation science field a novel assessment and solution-generating process that differs from previously cited approaches that outline specific responses to specific challenges [15-18].

This sense-making process is best suited for application when certain pre-conditions are present. First, research team leadership, such as supervisors and principal investigators, must be clear that implementation adaptations are expected due to the emergent nature of complex research settings. Second, team leaders must develop a research environment that harnesses creativity and encourages spontaneous emergent solutions, bounded by some control mechanisms (*e.g.*, the protocol). Lastly, to encourage and elicit creativity, leaders must empower all research team members to participate. Each team member’s voice should be explicitly valued, allowing members to feel that their contributions about real-time implementation challenges are welcomed and considered carefully by the team leadership. Teams that are unable to create these pre-conditions will not have high quality sense-making and will be at risk for misdiagnosing the challenge and its impact on the research study [4].

### Why a tool for evaluating research implementation challenges?

The TECH guides a research team to systematically assess the impact of the challenge and potential solutions that arise in the implementation of research while protecting research fidelity. Some challenges, if not addressed, will impact aspects of research fidelity [18], such as delivery, receipt of treatment, or enactment of skills (see Table 3 for definitions of aspects of fidelity). The challenge itself, therefore, might reduce study fidelity if not addressed. Solutions to address these challenges may reduce research fidelity through changes to the research design, research staffing, or research implementation procedures potentially impacting aspects of research fidelity such as design, training, and delivery [18]. The TECH provides a systematic process to ensure the fullest protection of study fidelity by asking questions throughout the process. For example, asking if the challenge threatens fidelity will help assess the urgency with which it should be addressed. The solution to the challenge is then developed in a manner to most fully protect study fidelity. Again, the extent to which fidelity is impacted is assessed. Challenges occur in unexpected and unavoidable ways. Ignoring them will sacrifice fidelity in that participants will not fully receive the intervention and enact new skills. Thus implementation challenges must be addressed, and TECH assists the team to address them systematically and in full regard for research fidelity. Further, TECH will guide the team to document changes so that any impact of the changes to the research design, training, and research implementation on results can be assessed.

**Table 3:** Aspects of fidelity in intervention research

**Aspect of Fidelity**[18]	**Examples of Issues considered**
Design	· Is the design consistent with the study theories?
	· Are intervention protocols standardized to a specified dose (*e.g.*, number, frequency, and length of contact)?
	· Is there a procedure specifying how to handle deviations from the specified treatment condition?
	· Is there a procedure for recording related issues in the study database?
Training	· Is there a standardized training protocol that specifies how interventionists are to be trained?
	· Is there separate training for interventionists delivering different treatment conditions?
	· Is there a plan for training new research team members?
	· Is there refresher training if there is more than one wave of recruitment?
Delivery	· Is there a procedure to ensure that interventions are delivered as intended?
	· How will intervention dose be tracked and recorded in the study database?
Receipt of Treatment	· Is there a procedure to measure participant adherence and behavior change?
	· Is there a procedure to measure change in knowledge level?
	· Is there a procedure for recording receipt-related data in the study database?
Enactment of Skills	· Is there a procedure for systematically assessing participants’ use of new behaviors?
	· Is there a procedure for recording enactment-related data in the study database?

Sense-making is appropriate for considering challenges in complex adaptive systems because the sense-making approach matches characteristics of a complex adaptive system in that it is a nonlinear, interactive, relationship-focused process [10]. Additionally, Jordan *et al.*[4] suggest that a focus on conversation and dialogue allows for effective sense-making among people with diverse perspectives. This approach is also used to develop team capabilities for effectiveness in dynamic situations [19]. Thus, our sense-making approach fits with the dynamic situations involved in our research implementation process across multiple sites.

We used complexity science principles to overcome challenges and develop solutions. Not surprisingly, we observed that the characteristics of complex adaptive systems, such as self-organization and co-evolution, were present as the research setting, research team, protocol, and site interacted with one another. For example, when clinical demands prevented many nursing home staff from participating in study activities, some nursing home managers responded by adjusting the staff assignments (*i.e.*, change in the research environment). Simultaneously, the research team responded by offering activities more frequently so that fewer staff needed to be removed from the floor for participation at any given time. On evening or night shifts, we held CONNECT sessions at the nursing stations to accommodate staff who could not leave the floor. Thus, as the research setting, research team, and research protocol interacted and challenges emerged, solution strategies also emerged. The sense-making process developed informally, but over time we found it useful to codify this process to systematize the approach to new challenges.

### Description and application of the sense-making process

We describe the processes codified in the TECH in a series of steps, even though in practice these are interactive and do not occur strictly stepwise. We discuss these as steps for sake of clarity. We then illustrate the application of the TECH to a few challenges our research team faced during implementation of the CONNECT study. Again, the TECH is most useful when the preconditions, described above, for effective sense-making are met.

### Identifying challenges

Those in direct contact with the research sites most often identified challenges that they suspected might impact successful implementation of the research design. Research team members in the field, such as research interventionists, were encouraged and rewarded for identifying challenges to successful implementation in research sites. The research interventionists identified challenges through activities such as noticing reluctance of staff to participate, listening to complaints of participants about research burden, asking questions of participants and site managers, and by describing patterns of participation and how these vary across the day. Others on a research team also identified challenges through activities such as regular review of field notes by the project coordinator, standard reports in team meetings that encouraged the interventionists to report challenges encountered, and regular (*e.g.*, quarterly review sessions) in which the team summarized challenges experienced and solutions implemented to date. Our core implementation team, including the principal investigators, co-investigators, project directors, and research interventionists, met in team meetings weekly and engaged in these activities. Members of the larger research team were involved as needed as described below.

### Interpreting the challenges

In weekly research team meetings, the core implementation research team engaged in discussion during which all members asked questions, verified the meaning, shared perspectives, and came to a clearer and shared understanding of the challenge and what it meant for the study. In these discussions, we incorporated the ideas of all research team members to interpret and diagnose the issues raised. Research interventionists would return to the research sites to ask clarifying questions of participants or other on-site research team members or remote team members would be involved through conference calls. Through such discussion, the research team members developed interpretations of the challenge, that is, we made sense of it, and developed a new understanding of the problem. When more than one challenge was raised, we prioritized the issues, giving priority to challenges thought to have the biggest impact on fidelity followed by those occurring most frequently.

**Figure 1:**
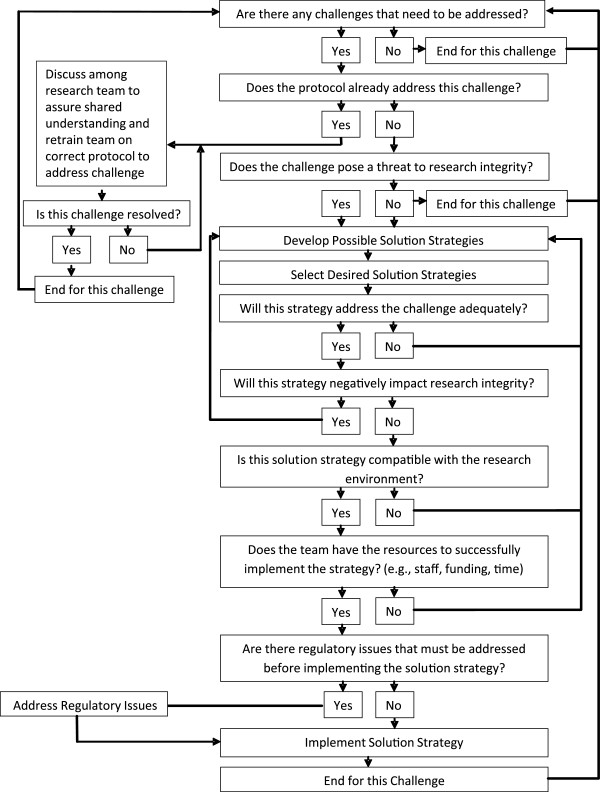
**Assessing implementation challenges and developing solution strategies.** Detailed flow diagram to guide systematic assessment of challenges and development of solutions.

In Figure 1, we describe the process used to assess the challenge and its meaning for the research study and later to develop solution strategies. Although the process itself is iterative, it is clearer to envision the steps as a linear algorithm. During the interpretation phase, as a team, we asked a series of questions about each challenge such as: Does the study protocol already address this challenge? Does the challenge pose a threat to research integrity? Asking these facilitated the research team to use a systematic process to assess the meaning of each challenge for the research design, the research design, research staffing, or research implementation. If we identified a challenge for which we thought we had an existing protocol, we discussed the protocol among the core implementation team to assure shared understanding and to reassess the adequacy of the protocol for addressing the challenge. Often in these instances, it was necessary to retrain team members on implementing the correct protocol to address the challenge.

### Generating and evaluating solution strategies

The process of interpretation and sense-making helped the team to generate ideas for solution strategies. Here again, open dialogue among the core implementation team members was required and individual core team members would also reach out to participants or research team members not in the core implementation team to gather ideas for solutions. These ideas were discussed in the core implementation team meetings; other research team members joined the discussion by conference call, if warranted. Once potential solution strategies were compiled, the research team considered whether or not the proposed solution(s) would address the challenge, support integrity of the research design, and improve the ability of the research team to implement the research protocol successfully. We used the algorithm (Figure 1) to ensure continued systematical evaluation of the various solution strategies. We asked questions including: To what extent will proposed solutions eliminate the challenge? Will the new strategy negatively impact research integrity? Is the solution strategy compatible with the research setting? Does the team have the resources to successfully implement the new strategy (*e.g.*, staff, money, time)? What regulatory issues must be addressed before implementing the new strategy? Discussing these questions provided a structure for evaluating our research team’s responses to implementation challenges.

### Addressing regulatory issues and implementing solution strategies

During deliberations of the research team, we identified and outlined any additional actions that were needed to accompany the proposed change to maintain research integrity. After a solution strategy was identified and approved by the research team, the research team adopted the change. This change was documented in the research protocol audit trail, research team meeting notes, and was communicated clearly to all research team members. Additional training was provided to team members when appropriate. When necessary, amendments were made to the research protocol and submitted for approval by the institutional review board. Any changes in the protocol where then subjected to the same process of review weekly in research team meetings to assess if the challenges persisted or if new challenges arose.

Overall, the TECH guides the team in an iterative process, requiring constant vigilance and communication regarding implementation challenges until the intervention research has come to a close. The TECH provided a standard process to allow for thoughtful change in the intervention protocol in response to key challenges that emerged in the clinical site(s). The high-level rules contained in the algorithm can be considered minimum specifications for research quality, providing direction pointing, boundaries, resources, and permissions for addressing unexpected challenges [8]. The semi-structured nature of the TECH allows for the application of these high-level rules without sacrificing the creative contributions of the research team members, whose experiences and perspectives are informed by different personal histories, idiosyncratic experiences, and varied levels of relevant content knowledge.

### Applying the tool for evaluating research implementation challenges

The following examples describe how we used the sense-making protocol codified in the TECH in actual challenges encountered in the study. In each nursing home site, there were some staff members who wanted to attend but were ultimately unable to participate in the scheduled 30-minute CONNECT class sessions because schedules would not permit. The research interventionists raised this challenge at several weekly research team meetings during which the team asked questions of each other to explore all aspects of the challenge and to ensure a shared understanding. In addition, the research interventionists asked questions when they returned to the sites to get additional information to help us to effectively interpret this challenge.

Once the challenge was clearly described for the research team, we worked through the steps in Figure 1 to further interpret the impact of the challenge. First, we examined whether or not the research protocol already addressed this challenge; that is, did the challenge emerge because of incorrect implementation of the existing protocol? In this case, we did not have an existing protocol to address the challenge, and it did not arise from incorrect implementation. In exploring the impact of this challenge on study fidelity, it was clear that the challenge would impact fidelity in terms of delivery, receipt of treatment, and enactment of skills (see Table 3). We deemed this a high priority issue because research integrity would be compromised by low attendance, and we concluded that the intervention dose would be reduced below levels that were necessary to maintain treatment fidelity.

The research team proceeded to brainstorm and consider potential strategies to address the challenge. One strategy identified involved delivering the CONNECT session with participants one-on-one in a condensed 15-minute format that we dubbed ‘mini-CONNECT.’ This approach was judged to be appropriate for the research setting because it did not impede clinical care and it could occur when the individual was available. Because the research interventionists were already on site, no additional resources were required for implementation. Finally, the overall integrity of the research was not compromised by the approach because the same core material was delivered to the staff in both the full length and ‘mini-CONNECT.’ The weakness of this approach was that because it was one-on-one, the participant would not benefit from the group learning that occurred in the regular CONNECT learning session. Thus the solution had a minor impact on delivery fidelity, but this impact was deemed to be far less than the greater impact on fidelity should we not address the challenge. This solution was implemented by the research team; a new protocol was developed and approved by the Institutional Review Board (IRB). The study database was modified to track how each subject received the delivery, either through the regular group learning session or the one-on-one format. Tracking delivery will allow us to explore whether this difference in delivery impacted study outcomes. This ‘mini-CONNECT’ example illustrates how a solution arises from the interaction of research interventionists and staff in the nursing home, ultimately requiring a change in the intervention protocol and data collection procedures.

Other examples of challenges encountered by our team and worked through the TECH include scheduling sessions to capture staff on all shifts and mitigating research interventionists’ fatigue. In the former, we were initially informed by administrators that night staff would be able to attend dayshift CONNECT educational sessions. In reality, night staff did not attend the sessions. To address this challenge, the team worked together to develop a solution strategy. The team ultimately chose to add additional CONNECT sessions late at night and early in the morning to improve rates of participation among night staff. This solution did not impact fidelity, but it had the potential to fatigue the research interventionists.

Related in part to this change in scheduling of sessions was the challenge of research interventionist fatigue. Research interventionists noted difficulty in staying motivated to work at a high level with up to three days of weekly travel to the same site. Additionally, research interventionists felt they needed two people on site to be successful in data collection activities. After employing the TECH tool to address this challenge, we rotated schedules for research interventionists to allow an occasional week with no travel. Additionally, we began to send pairs of research interventionists to research sites to facilitate data collection with less frustration and fatigue.

## Discussion

Challenges inevitably arise during any protocol implementation process [2,4], but researchers implementing multi-component interventions aimed at system change are likely to face both a greater number and more nuanced challenges than those implementing individual-focused interventions. When challenges arise, managing the tension between intervention fidelity and real life feasibility plays a key role in assessing challenges and developing solution strategies.

Although fidelity between the study protocol and implementation is necessary [20-22], systems level interventions must be developed with an awareness of the need for some flexibility in their delivery. Jordan *et al.*[2] suggest that linear implementation and inflexible fidelity protocols do not fit very well when implementing system change interventions, which by definition are shaped by nonlinear interaction, self-organization, and co-evolution of the complex adaptive system. Accordingly, it may be more useful to consider research implementation more fluidly, utilizing a sense-making lens [2]. A sense-making perspective provides a means to address challenges that emerge during research implementation in complex settings—an approach that allows for adaptation to the research setting while maintaining fidelity of the intervention [2]. McDaniel *et al.* [2, p. 193] state that ‘research design is not a prescription that defines what to do when but rather is the development of tentative guides for action.’ Instead of a rigid design, Beinhocker [23] proposes it is better to consider the spectrum of possibilities that may occur [24]. During the implementation of the CONNECT study, our research team learned that a systematic application of a sense-making perspective effectively guided our research team discussions to identify and implement appropriate solutions, and embodied Beinhocker’s (2006) guidance to move away from rigid research design.

The TECH provides a standard process with key questions to consider when challenges emerge—it focuses on sense-making as a process as opposed to a single decision event [18]. It is also best suited for research teams that embrace communication strategies such as trust, responsiveness, listening, paying attention, suspending assumptions, and ability to deal with misunderstandings, because these enhance sense-making [4]. The challenges encountered may be unexpected, but this protocol provides a systematic process for handling emergent challenges across varied research settings.

In any healthcare or non-healthcare setting, the leaders of the research team are key to successfully using the TECH tool by empowering all team members and encouraging open exchange of ideas. When the leaders value the voices of all of the research team members, it allows for emergence of richer information for interpreting the issues and forming solutions. Useful information might come from anyone on the team or it might emerge when processing divergent views to create a new idea not originally held by any team member. Specific to implementation challenges, it is imperative that all team members feel their voices are valued; if not, they may not share their implementation concerns with the team, potentially resulting in a negative impact to overall research integrity. Without team empowerment, members of the field team may implement emergent solutions without dialogue with the team, leading to protocol fidelity problems and potential negative impacts on overall findings. Alternatively, non-empowered team members may choose not to bring implementation issues to the team and instead continue to implement a flawed protocol. Thus, a team environment that encourages empowerment, open dialogue, creativity, and group problem-solving using the TECH will potentially strengthen both the protocol and research findings.

Another issue to consider when using the TECH is that each research team can adapt it to include or exclude relevant questions for their own research endeavors. The processes proposed in TECH can be incorporated into a research design, to ensure scientific rigor, flexibility, and adaptation to complex research settings. By using a standardized process for assessing research challenges within the team setting, the evolution of a study throughout an implementation period can be consistently documented. A standardized process, such as TECH, is also helpful when engaging a large research team—members are able to more effectively contribute feedback in a group setting or via distance by providing input within the framework provided by TECH. Lastly, as encouraged by scholars [2,3,24], this approach promotes the continuous improvement and evolution of the research process throughout the duration of the study.

The TECH is applicable across the research spectrum, from clinical trials to community based participatory research. At one extreme, clinical trials implemented without any adaptation for fear of contaminating the process may result in research outcomes that yield no real world applicability (*e.g.*, poor feasibility, inadequate delivery). These trials would benefit from the TECH because it provides a mechanism for maintaining fidelity of the intervention and study design, allowing for the development of more robust and feasible interventions in a live setting. At the other end of the continuum, community-based research may incorporate more stakeholders throughout the process allowing for better anticipation of potential challenges; however, because these are complex adaptive systems, it is not possible to anticipate all challenges occurring across diverse sites and over time, and the TECH provides a method for addressing additional unexpected challenges that arise.

Limitations to the TECH approach should be considered. As previously stated, this TECH may not work well in an environment where all team members do not feel empowered to participate fully; when faced with this issue, we encourage research teams to focus on building a core implementation team at a minimum and work to develop a culture of trust within that team. As with any protocol that provides rules and guidelines, there is a risk that creativity might be hindered; however, we argue that the sense-making evaluation protocol allows for responsible creativity, which balances the need for intervention fidelity and flexibility. Use of TECH may extend timelines because some solution strategies may also require additional IRB review. However, we believe that in the long run this may reduce time wasted by implementing protocols that are not functioning in the field.

Aspects of validity and reliability of the TECH were assessed during implementation of our pilot study; however, further testing is warranted. The pilot study implementation was successful, and thus the protocol exhibited face validity, helping us to successfully overcome implementation challenges in the field throughout the two pilot studies (VA and community studies). Other indicators of TECH reliability include that we met all deliverables in the study timelines, met recruitment goals, were able to consistently apply the same process of change to two studies, and retained the same research interventionist across the two pilot studies and the large extension study. Focus groups conducted following the interventions in both studies indicated that the interventions were well received; indirectly, this might indicate that we made appropriate adaptations to keep the intervention feasible and acceptable to the target audience. Overall, the TECH process both proved useful and was generally well received; members of the research team believe it facilitated useful adjustments to study implementation. We secured funding for a large extension study, and TECH has been helpful in addressing issues in an additional 16 nursing homes. We have observed, however, that because of the refinements to the study implementation protocols made using the TECH during the pilots, fewer adjustments have been required in the extension study, further supporting the TECH as a valid process.

We suggest, therefore, that the TECH tool is especially helpful in pilot studies in which the goal is to identify implementation challenges and refine intervention protocols for full-scale implementation. We believe, however, that it remains useful in subsequent research that uses the same intervention protocol because each research site is unique and will present new implementation challenges. Clinical practice changes over time, which can affect the research protocol implementation. New regulations impacting research might be enacted. Because these settings are complex adaptive systems means that study challenges inevitably arise in clinical research despite careful pilot work.

## Conclusions

In this paper, we have described our experience navigating the implementation of a multi-faceted research project in complex organization. Throughout project implementation, the intersection of our research site(s), research team, and intervention protocol constituted its own complex adaptive system. As such, we drew on complexity science principles to codify the TECH. We suggest that the TECH is appropriate for any research implementation endeavor, particularly for research in complex settings including nursing homes, hospitals, physician practices, clinics, and public health settings.

## Abbreviations

TECH: Tool for Evaluating Research Implementation Challenges.

## Competing interest

All authors declare that they have no competing interests.

## Authors’ contributions

RAA and CCE wrote the study protocols, acted as Dual PIs during implementation of the study, drafted sections of the manuscript, provided senior-level conceptual feedback on manuscript preparation and RAA revised the final manuscript. KMS acted as a project director during study implementation, wrote the first draft of the manuscript. KP acted as a project director during study implementation, drafted sections of the manuscript, and participated in manuscript revisions. EM acted as Co-I during implementation of the study and provided senior-level editorial feedback on manuscript preparation. AS acted as a research interventionist during study implementation—including delivery of the intervention and data collection, completed literature review activities to contribute to manuscript preparation, and provided editorial feedback on manuscript drafts. KAD acted as a research interventionist during study implementation—including delivery of the intervention and data collection, completed literature review activities to contribute to manuscript preparation, and provided editorial feedback on manuscript drafts. All authors read and approved the final manuscript.
